# Genome-Wide Association Study (GWAS) for Freezing and De-Acclimation Tolerance in Polish Winter Barley

**DOI:** 10.3390/ijms27062759

**Published:** 2026-03-18

**Authors:** Ipsa Bani, Santosh Hadagali, Magdalena Wójcik-Jagła

**Affiliations:** 1 Department of Plant Breeding, Physiology and Seed Science, University of Agriculture in Kraków, Podłużna 3, 30-239 Krakow, Poland; ipsa.bani.sd@student.urk.edu.pl; 2 Department of Plant Biology and Biotechnology, University of Agriculture in Krakow, Al. Mickiewicza 21, 31-120 Krakow, Poland; santosh.hadagali@student.urk.edu.pl

**Keywords:** freezing tolerance, de-acclimation tolerance, genome-wide association study (GWAS), mixed-linear model (MLM), single nucleotide polymorphism (SNP), diversity arrays technology sequencing (DArTseq)

## Abstract

Winter survival in barley depends on freezing tolerance and de-acclimation tolerance, yet their genetic determinants under increasingly unstable winters remain poorly understood. Here, 188 Polish barley accessions were evaluated over two consecutive growing seasons (2021–2022) using genome-wide association studies (GWAS) with a mixed-linear model (MLM) and high-density single nucleotide polymorphism (SNP) and diversity arrays technology sequencing (DArTseq) markers. Freezing and de-acclimation tolerance were quantified by 16 chlorophyll fluorescence parameters and post-freezing survival rates in plants subjected to 21 days of cold acclimation (4 °C/2 °C, day/night) and 7 days of de-acclimation (12 °C/5 °C, day/night). The results showed that freezing and de-acclimation tolerance are related but genetically distinct. The cold-acclimated (CA) state exhibited significant marker–trait associations on chromosomes 2H and 6H, whereas the de-acclimated (DA) state displayed a broader, more complex genetic architecture, particularly on chromosomes 2H and 7H. F_v_/F_m_ showed the strongest associations for both SNP and DArTseq markers in both states. PI_(csm)_, followed by PI_(cs0)_ and PI_(total)_, showed high SNP associations in the DA state, indicating a strong relationship between photosynthetic performance and freezing tolerance after de-acclimation. Notably, the DArTseq marker 11400277 on chromosome 7H showed multiple marker–trait associations across both states. These findings provide a genomic basis for marker-assisted selection of climate-resilient winter barley cultivars.

## 1. Introduction

Barley (*Hordeum vulgare* L.) is one of the major and earliest domesticated crops belonging to the family Poaceae. It is a vital staple for food, feed, and industrial applications. It is a rich source of high fiber, vitamins, and mineral content, and has many health benefits. It mainly grows in temperate climatic regions, where low-temperature stress is a major limiting factor to productivity. Fluctuating climatic conditions can affect the plant’s survival and the yield stability of the crop. The adaptability of winter barley to diverse agro-ecological zones has made it a model species for studying abiotic stress tolerance, especially in temperate climates where environmental fluctuations due to global warming pose a significant challenge [1].

Freezing tolerance and de-acclimation tolerance are two distinct traits in barley, indicating that some cultivars exhibit high freezing tolerance but are still susceptible to de-acclimation [2]. These are essential adaptive traits that enable winter barley to survive and thrive under a wide range of harsh and unpredictable winter conditions [2,3,4].

Freezing tolerance is the ability of the plant to survive when the temperature drops below zero and is one of the most acknowledged components of winter hardiness. It is influenced by various factors, including genetic background and environmental conditions. For example, winter and facultative cultivars of barley show higher tolerance compared to spring cultivars [5]. It was also observed that different barley cultivars exhibit varying levels of freezing tolerance [5]. Specific physiological markers have been identified, such as chlorophyll fluorescence parameters including ET_0_/CS and F_v_/F_m_, as reliable indicators of freezing tolerance, as they reflect the functionality of the photosynthetic apparatus in the cold stress [2,6]. These physiological markers enable early screening of the barley cultivars and provide insights into the plant’s stress resilience at a cellular level. The studies identified that different barley genotypes exhibit variable percentages of crown survival at low temperatures, with significant differences in traits like leaf relative water content and glycine betaine content, which are essential for freezing tolerance [7].

Several genetic factors and QTLs have been identified as key contributors to freezing tolerance, such as *Fr-H1*, *Fr-H2*, and *Fr-H3*, located on chromosome 5H. These loci were linked to significant SNP associations that contribute to frost tolerance in barley and were identified by genome-wide association studies (GWAS) [8,9]. The studies identified that multiple QTLs were linked to freezing tolerance and photosynthetic acclimation in winter barley. In two-rowed barley (P44 population), specific markers were located on 2H, 3H, and 7H, while, for six-rowed barley (CaP population), the markers were mainly located on all the chromosomes except 2H [10].

The studies have shown that the genotypes that possess smaller xylem vessels and stomata tend to be more freezing-tolerant than freezing-sensitive genotypes, regardless of cold acclimation. Small stomatal length was also correlated with a smaller xylem vessel cross-sectional area. These anatomical traits may offer a stable selection target for breeding [11].

In contrast, de-acclimation refers to the decrease and loss of the freezing tolerance that was initially acquired through cold acclimation. In natural environments, this process typically occurs in early spring as the temperature rises [12]. De-acclimation is of two types: active and passive de-acclimation [4]. Active de-acclimation occurs during winter, a period when plants are expected to stay fully cold-acclimated [2]. Antioxidant enzymes and additional oxidoreductases are indicated to play an essential role in regulating active de-acclimation. Also, active de-acclimation is thought to be viewed as a chance to recover from stress [4]. On the other hand, passive de-acclimation occurs when freezing tolerance is highest during mid-winter and subsequently declines due to the cultivar depletion and efficient utilization of the organic reserves within the plant [13]. Passive de-acclimation is initiated mainly by developmental transitions such as the shift from vegetative to generative stages that involve complex changes in gene expression at diverse regulatory levels [3]. The key factor on which the kinetics of de-acclimation depend is temperature and duration of the warm period, as well as the cultivar [14].

De-acclimation tolerance is the ability of the plant to maintain the highest possible freezing tolerance even after de-acclimation [15]. It is influenced by various physiological, genetic, and hormonal factors, as well as the plant’s ability to manage the stress responses. It is also observed that the mutations in the genes responsible for the synthesis and response to brassinosteroids enhance the de-acclimation tolerance [16]. Recent studies show that microRNAs (miRNAs) play critical roles in regulating various physiological activities, such as cold response in plants. Novel miRNAs and their target genes have been identified in response to cold de-acclimation in barley, offering potential targets for enhancing the de-acclimation tolerance [17].

Plant breeding, marker-assisted selection (MAS), and genome-wide association studies (GWAS) are closely interconnected components of modern crop improvement strategies. Plant breeding seeks to enhance traits like yield, stress tolerance, and quality, but traditional methods often fall short when dealing with complex, polygenic traits influenced by environmental variability [18]. MAS addresses these limitations by using molecular markers linked to key traits, enabling earlier and more precise selection. It is particularly effective for traits that are difficult to phenotype, controlled by recessive alleles, or require pyramiding of multiple loci within a single genotype [18,19]. GWAS strengthens MAS by identifying genome-wide marker–trait associations across diverse germplasm. It thereby uncovers QTLs for important traits such as yield, disease resistance, and heading date in barley [20].

Genome-wide association mapping is a very powerful method used to associate specific genetic variations with a particular trait. It has been widely used for the genetic dissection of complex traits [21]. It enables the identification of genes of interest by screening large and diverse populations by integrating phenotypic observation with genome-wide genetic variations to uncover the genotype-phenotype relationship [22]. The GWAS model is influenced by various factors like linkage disequilibrium (LD), population density, and minor allele frequency (MAF) [20]. It is quicker and more precise than traditional linkage mapping, as it can map QTL at a significantly higher resolution in case more SNP markers are available [23].

Since molecular markers like SNPs and DArTseq offer high-throughput, cost-effective, and genome-wide coverage, they have become essential tools in identifying quantitative trait loci (QTLs) associated with freezing and de-acclimation tolerance in barley [10,24,25]. Amongst the most frequently used markers in GWASs, SNPs are widely applied due to their abundance and stability across the genome. DArTseq markers are also a valuable resource, providing complementary genome-wide information; DArTseq combines the advantages of both SNP and DArTseq markers, enabling even more detailed genetic analyses [26]. These markers facilitate marker-assisted selection (MAS) by allowing the breeders to identify genotypes carrying alleles associated with freezing tolerance in cold-acclimated state and de-acclimated state directly at the DNA level. This eliminates the need for repeated phenotypic screening under variable and unpredictable environmental conditions [27,28]. Marker-assisted selection provides the potential of assembling the target traits within the same genotypes more precisely, with fewer selection cycles and minimal genetic loss [29]. By integrating marker-assisted selection (MAS) and GWAS, plant breeders can make informed decisions based on the genotypic data, accelerating the development of improved barley varieties with multiple beneficial traits [30].

Despite advances in understanding the genetic basis of freezing stress responses in Polish winter barley, there remains limited knowledge about the comparative genetic architecture of freezing tolerance and de-acclimation tolerance. Therefore, our study aims to enhance understanding of these two distinct traits using GWAS analysis on a panel of 188 Polish winter barley accessions, with genotypic data from both marker types (DArTseq and SNP) and phenotypic data related to freezing and de-acclimation tolerance traits. We hypothesize that the genetic determination of freezing tolerance and de-acclimation tolerance differs. A comparative analysis will reveal distinct genetic factors controlling these traits. Ultimately, these findings will support plant breeding efforts and improve marker-assisted strategies aimed at developing climate-resilient barley cultivars.

## 2. Results

### 2.1. Genotyping

A total of 188 barley accessions were genotyped using DArTseq and SNP markers, which showed widespread polymorphism associated with freezing tolerance under CA and DA states. Initially, we obtained a total of 14,838 SNP markers and 30,591 DArTseq markers. To ensure the reliability of genotypic differentiation, markers with a polymorphism information content (PIC) higher than 18% were used as input data for genome-wide association studies (GWAS). After this quality filtering, 18,326 DArTseq markers and 10,752 SNP markers were retained for subsequent GWAS. GWAS was conducted using a mixed linear model (MLM) approach implemented in TASSEL software version5.2.188. Following the association analysis, markers were filtered based on a significance threshold of *p* ≤ 0.01, resulting in the identification of 2584 DArTseq and 2956 SNP markers associated with the traits under investigation. Specifically, for the DA state, 1426 DArTseq and 1767 SNP markers were obtained, while 1158 DArTseq and 1189 SNP markers were found for the CA state.

### 2.2. Phenotyping

To evaluate both freezing tolerance and de-acclimation tolerance in barley, 16 physiological measurements were observed across 188 accessions under two environmental states, i.e., CA and DA states. It included several chlorophyll fluorescence parameters and survival rate. The results varied significantly between the CA and DA states, and there was also considerable diversity in trait responses among the different barley accessions within each state (Table S1). The de-acclimation affected the measured chlorophyll fluorescence parameters, mostly causing a decrease compared to hardened plants. In 2021, the PI_(csm)_ and PI_(total)_ parameters decreased the most, followed by PI_(csm)_ in 2022.

De-acclimation also caused a decrease in plant survival rates. In 2021, survival decreased from 35.4% to 19.0%, and in 2022, from 23.1% to 4.9%. Regardless of freezing tolerance in the cold-acclimated state, a very large variation in freezing tolerance was observed in de-acclimated plants tested in both years.

### 2.3. Genome-Wide Association Mapping

#### 2.3.1. Marker Distribution Under Cold-Acclimated (CA) State

**Figure 1:**
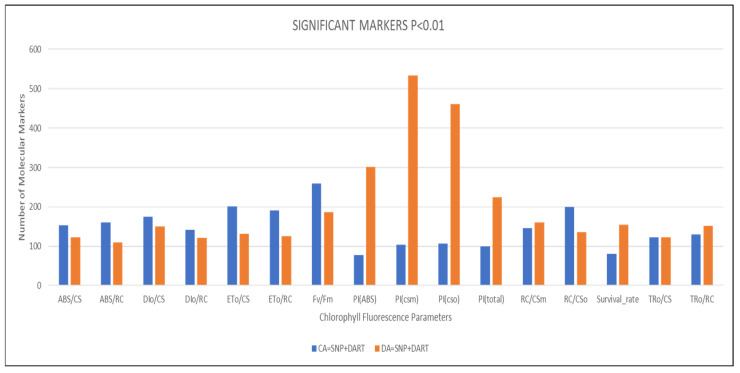
Combined number of makers associated with different chlorophyll fluorescence parameters in cold-acclimated (CA) and de-acclimated (DA).

For the CA state, the number of associated SNP markers varied widely across different phenotypic traits, ranging from 17 to 155. The highest counts were observed for F_v_/F_m_ (155), which indicates the maximum quantum efficiency of photosystem II, and RC/CS_0_ (134), representing the density of reaction centers per leaf cross-section. Additionally, both ET_0_/CS and ET_0_/RC parameters related to electron transport efficiency had equal SNP marker counts of 107. The lowest SNP counts were observed for PI_(csm)_ (17) and PI_(ABS)_ (21), suggesting either fewer genetic variants influencing these physiological parameters or lower marker coverage in these genomic regions (Figure 1).

**Figure 2:**
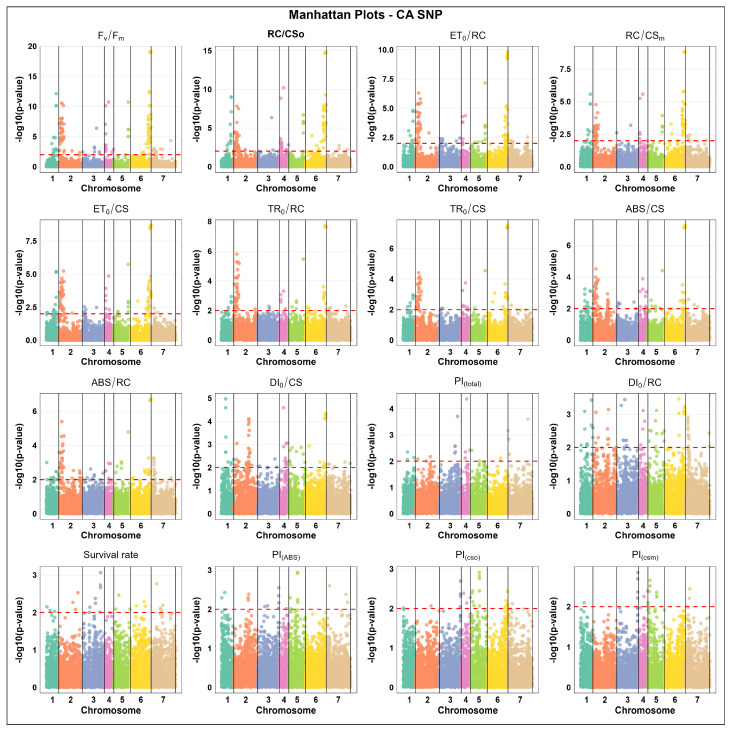
Manhattan plots of SNP marker associations under cold-acclimated (CA) states across 16 distinct physiological parameters, including chlorophyll fluorescence parameters and survival rate in winter barley. The x-axis represents the relative density and physical positions of the SNP markers mapped in the cold-acclimated (CA) state across the seven chromosomes of barley (1H–7H), while the y-axis shows the −log10 *p*-values for the association power for each marker–trait pair. The peaks above the red dashed line indicate significance thresholds of −log10 *p* ≤ 0.01 = 2.

The association analysis for SNP markers in CA state indicate that chromosome 2 carried the highest number of significantly associated SNP markers for key parameters such as RC/CS_0_ (44 markers), ET_0_/RC (44), TR_0_/RC (39), ABS/CS (40), and ABS/RC (38), thus highlighting its key role in freezing tolerance. In contrast, the lowest marker counts were generally found on chromosomes 3, 4, 5, or 7, depending on the parameters, e.g., TR_0_/CS displayed the lowest count on chromosome 7 (1 marker), PI_(cso)_ showed the lowest on chromosomes 1, 2, and 7 (1 each), and PI_(total)_ on chromosomes 5 and 6 (2 each). Chromosome 6 showed the highest associated marker count for F_v_/F_m_ (53). These findings suggest chromosome 2 is especially important for SNP marker associations in the CA state, while the other chromosomes contribute fewer significant markers for many physiological parameters (Figure 2).

Similarly, the number of CA-associated DArTseq markers ranged from 50 to 105, with F_v_/F_m_ again exhibiting the highest count (105), emphasizing its genetic complexity and importance in describing plant performance after freezing. High DArTseq marker counts were also observed in ET_0_/CS (94) and DI_0_/CS (89), reflecting significant loci involved in photosynthetic efficiency after freezing in cold-acclimated plants. Conversely, the lowest CA DArTseq marker counts were observed for TR_0_/CS (50) and RC/CS_m_ (52), which may indicate fewer polymorphisms or less pronounced genetic control of these traits under post-freezing conditions. Overall, these results demonstrate a heterogeneous distribution of genetic markers in the CA state (Figure 1).

**Figure 3:**
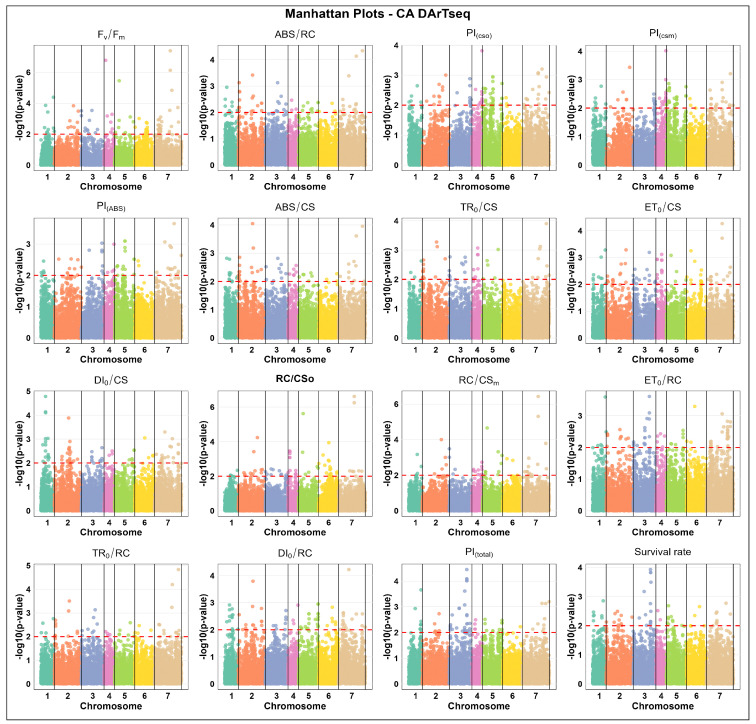
Manhattan plots of DArTseq marker associations under cold-acclimated (CA) states across 16 distinct physiological parameters, including chlorophyll fluorescence parameters and survival rate in winter barley. The x-axis represents the relative density and physical positions of the DArTseq markers mapped in cold-acclimated (CA) state across the seven chromosomes of barley (1H–7H), while the y-axis shows the −log10 *p*-values for the association power for each marker–trait pair. The peaks above the red dashed line indicate significance thresholds of −log10 *p* ≤ 0.01 = 2.

The results for DArTseq markers associated with freezing in the CA state indicate that the chromosome with the highest number of significant markers varied by trait. For instance, chromosome 7 contains the most markers associated with F_v_/F_m_ (42 markers) and ET_0_/RC (37), and chromosome 5 showed the highest number of associated markers for PI_(csm)_ (30) and PI_(cs0)_ (28), while chromosome 2 was rich in markers associated with DI_0_/CS (31). Conversely, the lowest marker counts were often found on chromosomes 4 or 6, such as for PI_(total)_ on chromosome 6 (1 marker), survival rate on chromosomes 4 and 6 (2 each), and PI_(cs0)_ and PI_(csm)_ on chromosome 6 (2 each). These patterns indicate that, unlike the CA SNP data, marker distribution in CA DArTseq is more trait-dependent, with different chromosomes showing the highest associations for specific traits in winter barley, and thus hinting at QTL existence in these regions (Figure 3).

#### 2.3.2. Marker Distribution Under De-Acclimated (DA) State

Associated SNP marker counts under de-acclimated (DA) states varied widely, ranging from 36 to 435. The highest counts were observed for PI_(csm)_ (435), PI_(cs0)_ (359), and PI_(total)_ (137), highlighting strong genetic control over photosynthetic efficiency traits of DA plants. The lowest SNP counts were recorded for ABS/RC (36), RC/CS_0_ (42), and ET_0_/RC (42) (Figure 1).

**Figure 4:**
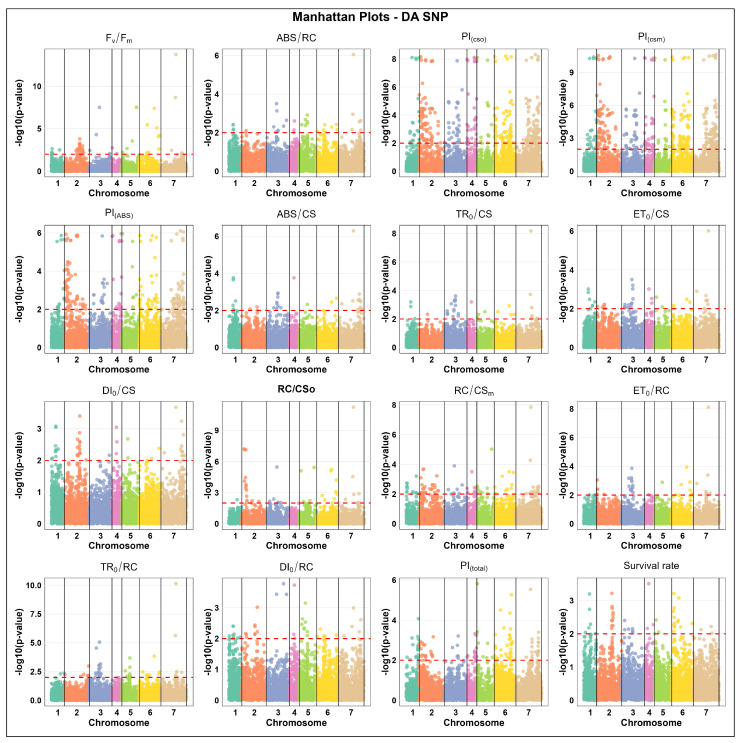
Manhattan plots of SNP marker associations under de-acclimated (DA) states across 16 distinct physiological parameters, including chlorophyll fluorescence parameters and survival rate in winter barley. The x-axis represents the relative density and physical positions of the SNP markers mapped in a de-acclimated (DA) state across the seven chromosomes of barley (1H–7H), while the y-axis shows the −log10 *p*-values for the association power for each marker–trait pair. The peaks above the red dashed line indicate significance thresholds of −log10 *p* ≤ 0.01 = 2.

The association analysis for DA SNP markers showed that the chromosome displaying the highest number of significant markers was usually chromosome 2 or 7 for most parameters. Chromosome 2 exhibited the highest counts for F_v_/F_m_ (33 markers), PI_(csm)_ (112), PI_(ABS)_ (72), and RC/CS_m_ (29), while chromosome 7 showed the highest number of associated markers for PI_(cs0)_ (95), PI_(csm)_ (104), and ABS/CS (21). In contrast, the lowest marker counts for each parameter were commonly found on chromosomes 4 or 5, such as ABS/CS on chromosomes 4 and 5 (1 marker each), TR_0_/CS on chromosomes 2 and 4 (1 each), and ET_0_/RC on chromosomes 4 and 5 (1 each) (Figure 4).

Meanwhile, the associated DArTseq marker counts ranged from 67 to 123, with the highest values found for F_v_/F_m_ (123), PI_(cs0)_ (102), and PI_(csm)_ (99). The lowest DArTseq counts were associated with RC/CS_m_ (68) and ABS/CS (81) under the DA state (Figure 1).

**Figure 5:**
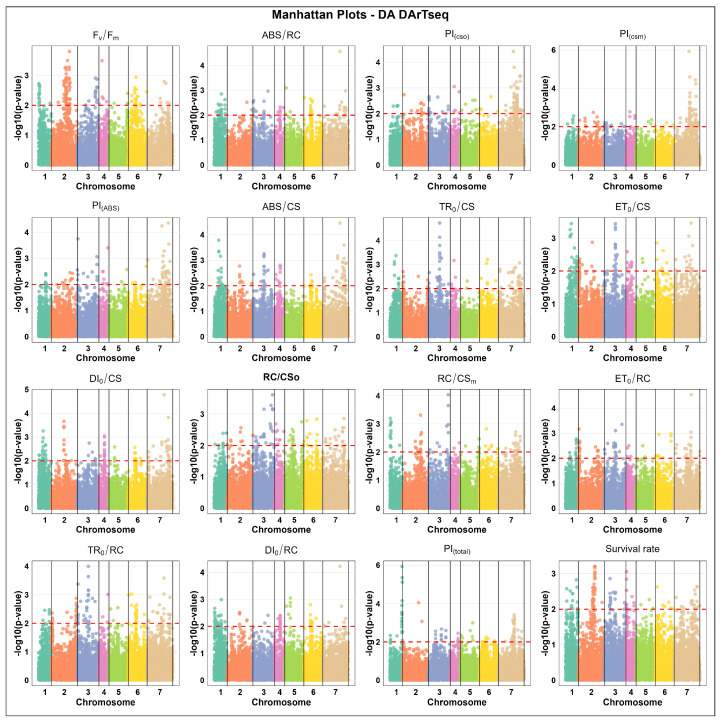
Manhattan plots of DArTseq marker associations under de-acclimated (DA) states across 16 distinct physiological parameters, including chlorophyll fluorescence parameters and survival rate traits in winter barley. The x-axis represents the relative density and physical positions of the DArTseq markers mapped in de-acclimated (DA) state across the seven chromosomes of barley (1H–7H), while the y-axis shows the −log10 *p*-values for the association power for each marker–trait pair. The peaks above the red dashed line indicate significance thresholds of −log10 *p* ≤ 0.01 = 2.

The association analysis for DArTseq markers related to the response to freezing in the DA state also showed that the highest marker counts appeared on chromosome 7 or chromosome 2, depending on the trait. Chromosome 7 exhibited the highest number of significant associated markers for PI_(cs0)_ (58 markers), PI_(csm)_ (61), PI_(ABS)_ (34), ABS/CS (30), and PI_(total)_ (31), while chromosome 2 displayed the most associated markers for F_v_/F_m_ (58) and survival rate (37). In contrast, the lowest counts were frequently observed on chromosomes 3, 4, or 5—for example, PI_(total)_ on chromosome 2 (2), PI_(ABS)_ and TR_0_/CS on chromosome 5 (2 each), RC/CS_m_ on chromosome 4 (2), and DI_0_/RC on chromosome 3 (2) (Figure 5). These results show consistency of the SNP and DArTseq marker systems in the case of uncovering the genetic determination of freezing response in the DA state.

#### 2.3.3. Chromosome Distribution of Associated Markers in CA and DA

- *In the cold acclimated (CA) state*

**Figure 6:**
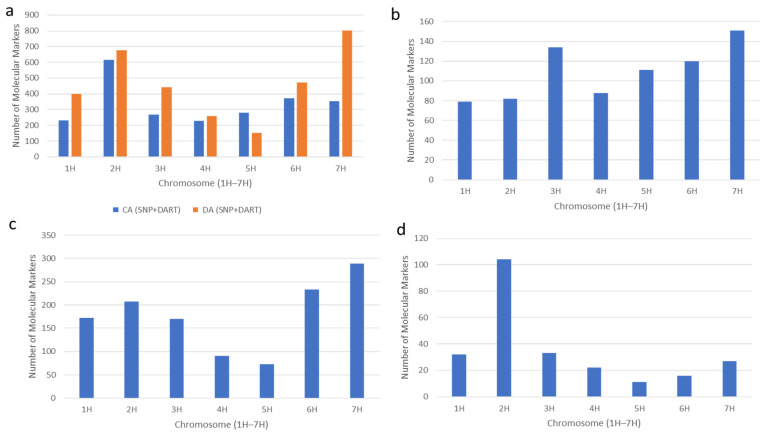
Number of Molecular Markers and their chromosome-wise distribution, (**a**) Marker–trait association in cold-acclimated (CA) and de-acclimated (DA) state, (**b**): Number of genetic markers uniquely associated with freezing tolerance in cold-acclimated (CA) state, (**c**) Number of genetic markers uniquely associated with freezing tolerance in de-acclimated (DA) state, (**d**) Number of common genetic markers associated with freezing tolerance in both cold-acclimated (CA) and de-acclimated (DA) states.

The CA SNP marker association counts per chromosome ranged from 81 to 414. The highest number of SNP markers (414) was mapped on chromosome 2H, followed by 6H with 290 and 4H with 112. The lowest SNP count was observed on chromosome 3H with 81 markers. Meanwhile, the DArTseq markers CA counts per chromosome ranged from 83 to 268, with chromosome 7H showing the highest count (268), followed by 3H (187) and 5H (182). The lowest DArTseq marker count was observed on chromosome 6H with 83 markers. This distribution indicates that chromosomes 2H, 6H, and 7H harbor key genomic regions controlling barley’s adaptive response to freezing tolerance in the CA state (Figure 6a).

- *In the de-acclimated (DA) state*

The SNP marker association counts per chromosome in the DA state ranged from 88 to 445. The chromosome 2H exhibited the highest count (445), followed by 7H (402) and 6H (312), with chromosome 5H displaying the lowest count of 88 markers. On the other hand, the DArTseq markers’ association per chromosome ranged from 63 to 395, with the highest counts on chromosome 7H (395) and chromosome 1H (232), which was tied with chromosome 2H. Similarly to SNPs, the lowest associated DArTseq count was observed on chromosome 5H with 63 markers. These findings emphasize the significant role of chromosome 7H in harboring genetic factors influencing de-acclimation response, with chromosomes 2H and 6H also contributing substantially, underscoring their importance in breeding barley cultivars with enhanced de-acclimation tolerance (Figure 6a).

**Figure 7:**
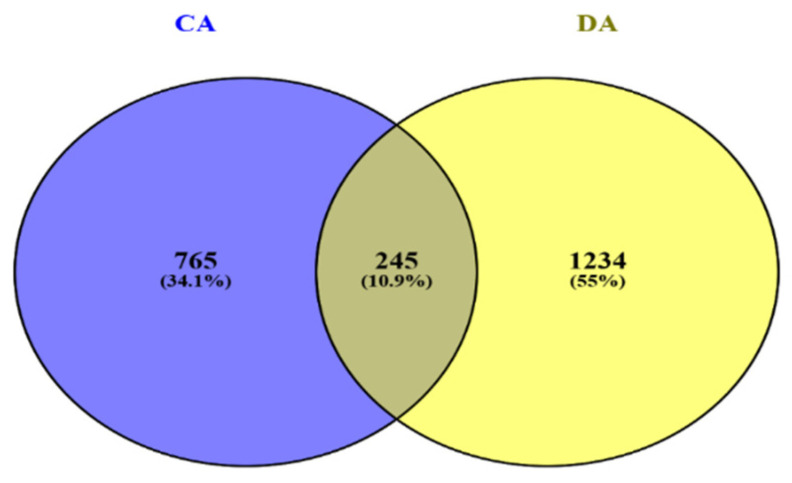
Venn diagram illustrating the distribution of makers unique to the cold acclimated (CA) state, unique to the de-acclimated (DA) state, and common to both the CA and DA states.

The Venn diagram for the MLM results illustrates the distribution of the genetic markers associated with freezing tolerance in CA and DA states in winter barley. A total of 765 markers (34.1%) were uniquely associated with CA, while 1234 markers (55%) were associated only with DA. Additionally, 245 markers (10.9%) were common to both the CA and DA states, indicating shared regions involved in reaction to freezing in both the CA and DA states. The higher number of unique markers in DA suggests that freezing tolerance in de-acclimated plants involves a broader or more diverse set of genetic factors compared to freezing tolerance after cold acclimation in the studied group of Polish winter barley breeding lines (Figure 7).

The number of markers unique to the CA state per chromosome ranged from 79 to 151. The highest number of markers associated with freezing tolerance-related traits in the CA state was 151 on chromosome 7H, followed by 3H with 134 and 6H with 120. The lowest count appeared on chromosome 1H with 79 markers, implying a lesser role in determining the physiological traits specific to freezing tolerance in the CA state (Figure 6b).

For the DA state, the number of markers uniquely associated with freezing tolerance per chromosome ranged from 73 to 289. Chromosome 7H displayed the highest number with 289 markers, followed by 6H with 233 and 2H with 207. The lowest count was found on chromosome 5H with 73 markers (Figure 6c). The result shows that the genetic determinants of traits underlying de-acclimation tolerance were unevenly distributed on the chromosomes.

Among markers common to both the CA and DA states across barley chromosomes varied from 11 to 104. The chromosome 2H exhibited the highest number of common markers with 104, highlighting its significant role in regulating traits relevant to freezing tolerance across both states. The remaining chromosomes contained substantially fewer commonly associated markers, ranging from 33 on chromosome 3H to only 11 on chromosome 5H (Figure 6d).

Several of the common markers associated with the traits underlying freezing tolerance in both the CA and DA states were selected due to their possible usefulness in plant breeding. Out of all, the markers that were associated with at least three chlorophyll parameters indicating the damage to the photosynthetic apparatus after freezing in the DA state, and with survival rate after de-acclimation, were selected. Similarly to the DA state, we also verified the traits associated with these markers in the cold-acclimated (CA) state. Nearly all the selected markers were DArTseq markers, predominantly located on chromosomes 1H and 7H; there was also one located on 2H. The only SNP marker that met the selection criteria was located on chromosome 4H (Table 1).

Among the associated chlorophyll fluorescence parameters, ABS/CS, ABS/RC, DI_0_/CS, TR_0_/CS, and TR_0_/RC were the most strongly represented. In contrast, the least represented parameters were PI_(ABS)_, F_v_/F_m_, and RC/CS_m_ in the DA state, and PI_(total)_, which was particularly state-specific, only appearing in the CA state.

The SNP marker 6437688 acted as a trait-association hotspot in the DA state, where it displayed a broader range of associations, while in the CA state, it was uniquely associated only with PI_(total)_.

Among all markers, the DArTseq marker 11400277 on chromosome 7H stood out as it was associated with the largest number of chlorophyll fluorescence parameters and also showed association with similar parameters in both DA and CA states. These selected common markers shared between CA and DA states were associated with the chlorophyll fluorescence parameters reflecting the photosynthetic efficiency, heat dissipation, energy absorption under stress, electron transport, and overall plant survival in DA states, particularly indicating damage to the photosynthetic apparatus through energy dissipated from PSII reaction centers and changes in the maximum quantum yield of photosystem II. Collectively, our results map the major genomic regions consistently linked both to freezing tolerance and de-acclimation tolerance, marking the most stable and reproducible loci across the CA and DA states. These markers represent valuable resources for breeder selection and provide promising targets for developing barley cultivars with enhanced resilience to increasingly fluctuating winter climate conditions.

## 3. Discussion

Understanding the genetic mechanisms underlying freezing tolerance and de-acclimation tolerance in cereals is essential for the development of cultivars resilient to increasingly variable winter climates. Although the genetic basis of freezing tolerance during cold acclimation has been extensively studied in barley [5,8,31,32], little information is known about how plants maintain freezing tolerance when exposed to short mid-winter warm spells, i.e., de-acclimation tolerance. To address this knowledge gap, we performed a comprehensive genome-wide association study (GWAS) on 188 winter barley genotypes to compare the genetic architecture of freezing tolerance under both CA and DA states. Using both SNP and DArTseq markers, marker–trait associations were identified through a mixed linear model (MLM) approach. Our GWAS analysis identified significant key marker–trait associations, with chromosomes 2H, 6H, and 7H showing major roles in both CA and DA states. This analysis revealed significant variation in freezing tolerance and de-acclimation tolerance across genotypes, enabling the identification of genomic regions and markers associated with these two adaptive traits. These findings provide new insights into the dual mechanisms governing winter survival in barley.

In our results, SNPs generated higher numbers overall, particularly for PI-related parameters, but both SNP and DArTseq consistently highlighted chromosomes 2H and 7H as major genomic regions under the DA state. Also, the results of our analysis reveal that chromosome 2H is foundational for the freezing tolerance, while chromosome 7H plays a critical role during de-acclimation tolerance, especially in maintaining the integrity of the photosynthetic apparatus and direct survival of the plants. These distinct but partially overlapping genetic architectures support our hypothesis that freezing tolerance and de-acclimation tolerance are independent traits contributing to a cumulative trait of winterhardiness. De-acclimation involves broader genetic complexity and physiological regulation [2]. This phenomenon might result from the fact that the Polish accessions used for this study are the result of years of selection towards freezing tolerance in a CA state, which has naturally decreased the genetic diversity of this trait in this particular genepool. On the contrary, the selection towards freezing tolerance in the DA state was not carried out at all, or at least not with premeditation.

Another key finding from our study is the substantial difference in the number and distribution of the markers associated with freezing tolerance between CA and DA states. A total of 765 markers (34.1%) were uniquely associated with the CA state, whereas 1234 markers (55%) were unique to the DA state, and only 245 markers (10.9%) were common to both the CA and DA states. Although both states showed the highest number of significant associations on chromosome 7H, the DA state exhibited a greater overall abundance of significant markers, indicating a broader and more complex genetic regulation of tolerance during de-acclimation. This suggests that while the key genomic regions influencing freezing tolerance overlap between the two states (CA and DA), the genetic responses activated during de-acclimation are more diverse and extensive. Moreover, these findings highlight a substantial breeding potential for improving de-acclimation tolerance in barley. On the other hand, the genetic diversity of responses to freezing in the CA state seems to be narrower, which was to be expected because the breeding process focused on this trait for years, limiting the genetic pool related to freezing tolerance in barley [33,34,35]. The shared marker distributions reflect the complexity of physiological traits controlling freezing tolerance. Among all chlorophyll fluorescence parameters examined, F_v_/F_m_ emerged as particularly informative because a higher number of markers were significantly associated with this parameter in the CA and DA states. F_v_/F_m_ quantifies the efficiency with which the absorbed light is converted into photochemistry, and a decrease in its value often indicates stress-induced damage to PSII [36,37]. F_v_/F_m_ measurements evaluated under field conditions were also found to be the most widely used chlorophyll fluorescence-related parameter, which is highly associated with freezing tolerance [2,38]. Similarly, PI_(csm)_ exhibited the highest number of associations with SNP markers in the DA state. Previous studies showed that PI_(CSm)_ is the foremost indicator for freezing tolerance in triticale [39,40].

Our results concur with earlier studies, which highlighted that key genomic regions, such as barley chromosomes 2H and 7H demonstrate the genetic basis of freezing tolerance, consistent with findings in barley [10] and comparative studies in winter canola and wheat [41,42]. Previous studies on barley identified eleven QTLs for the two-rowed barley population; among these, five QTLs were specifically associated with freezing tolerance and photo-acclimation, located on chromosomes 2H, 3H, and 7H. Whereas in the six-rowed barley population, only one QTL was linked to photo-acclimation, but ten QTLs were associated with freezing tolerance. These QTLs were distributed across all the chromosomes except 2H [10].

Our analysis revealed that, when considering only the genetic markers common to both the CA and DA states, chromosome 2H contained the highest number of loci associated with freezing tolerance. However, when the CA and DA datasets were analyzed separately (without restricting to common markers), chromosome 7H showed the highest number of markers associated with freezing tolerance in both states. Previous studies have identified the *Frost resistance-H2 (Fr-H2)* locus on chromosome 2H, which holds a major QTL affecting freezing tolerance in barley. This locus contains multiple C-Repeat Binding Factor (CBF) genes crucial for refiguring the plant transcriptome in response to low temperatures [43,44]. Also, a locus associated with frost tolerance in the reproductive tissues of barley was identified on chromosome 2H [45]. This further emphasizes its important role in freezing tolerance across different tissues and developmental stages of the plant. Similarly, the involvement of chromosomes 4H and 5H has been identified as a significant contributor to freezing tolerance or low temperature tolerance in barley [9,34,46].

Several common markers associated with the traits underlying freezing tolerance in both CA and DA states were observed in this study, highlighting their potential for breeding applications. Moreover, some markers identified in the present study were associated with both the chlorophyll fluorescence parameters, which indirectly inform us about freezing tolerance, indicating the level of damage to the photosynthetic apparatus after freezing. These markers were associated with the survival rate after de-acclimation, which is a direct indicator of plant freezing tolerance. Chlorophyll fluorescence parameters proved to be an effective tool for assessing freezing damage to the plants, as they accurately reflect damage to the photosynthetic apparatus, which is highly correlated with later plant survival [40,47,48,49]. The chlorophyll fluorescence traits most frequently associated with the selected markers in our study were ABS/CS, ABS/RC, Dl_0_/CS, TR_0_/CS, and TR_0_/RC. Similarly, the previous studies identified that QTLs associated with chlorophyll fluorescence parameters ABS/CS_0_, TR_0_/CS_0_, ABS/CS_m_, and DI_0_/CS_m_ on chromosome 6H, highlighting their genetic significance for photosynthetic efficiency in barley [50]. Also, recent studies have identified quantitative trait loci (QTLs) associated with these chlorophyll fluorescence parameters across various crops such as barley, maize, wheat, and soybean [47,48,50,51,52,53].

We found that rather than acting as single-trait associations, the majority of the markers selected in our study as most suitable for the breeding process were clustered on chromosomes 1H, 7H, and 2H. Only one SNP marker on chromosome 4H was significantly associated with key traits in winter barley, which are indirectly responsible for freezing tolerance, in both DA and CA states. Similarly, the previous GWAS on barley also showed a significant association with low temperature tolerance on 4H and 5H [9]. Among all the selected markers, the DArTseq marker 11400277 located on chromosome 7H was the most prominent hotspot, showing associations with the largest number of similar chlorophyll fluorescence parameters in both states, and thus was the most promising in MAS applications. The most valuable markers for breeders will probably be those that enable the simultaneous selection of freezing-tolerant plants in both CA and DA states within a single analysis. By focusing on the markers that are relevant in both states, breeders can select for traits that confer resilience to temperature fluctuations, thus enhancing crop survival rates under climate change conditions [54].

While our study successfully identified SNP and DArTseq markers associated with key traits under both CA and DA states, further investigation is required in order to draw more general conclusions. The current analysis was conducted on a region-specific experimental population, and environmental variation was limited to specific conditions of our experiment. Therefore, validation of the identified markers across a broader set of winter barley accessions, multi-environment trials, and diverse agroclimatic regions is essential to confirm their stability, reproducibility, and predictive accuracy.

Our results indicated that chromosomes 1H, 7H, and 2H (based on DArTseq markers) and chromosome 4H (based on SNP markers) harbor loci associated with freezing tolerance-based chlorophyll fluorescence parameters. Future studies should aim to integrate these validated markers into marker-assisted selection (MAS) pipelines and genomic selection models capable of accelerating the breeding of barley varieties with enhanced freezing tolerance and de-acclimation tolerance. Such integrative approaches will strengthen the practical application of these findings in breeding programs and contribute to the development of climate-resilient winter barley cultivars.

## 4. Materials and Methods

### 4.1. Plant Materials

A total of 188 Polish barley accessions used in this study were collected from various plant breeding companies, as listed in the previous study [55].

### 4.2. Phenotyping

Phenotypic data were collected for 16 physiological parameters related to freezing tolerance under cold-acclimated and de-acclimated states (de-acclimation tolerance), including chlorophyll fluorescence parameters (ABS/CS, DI_0_/CS, F_v_/F_m_, TR_0_/CS, TR_0_/RC, ABS, PI_(csm)_, PI_(cs0)_, PI_(total)_, RC/CS_m_, RC/CS_0_—the terms are clarified in Table S2 and survival rate, over two consecutive growing seasons (2021–2022). The plants were grown under controlled conditions following a protocol modified from the previous study [4]. After sowing, the pots were placed in a growth chamber in darkness at 25 °C/17 °C (day/night). When seedlings began to emerge, they were exposed to 12 h/12 h (light/dark) photoperiod with an irradiance of 400 µmol m^−2^ s^−1^ (HPS lamps, SON-T+ AGRO, Philips, Eindhoven, The Netherlands). Eight days after sowing, the temperature was reduced to 15 °C/12 °C (day/night) and maintained until the start of cold acclimation. Cold acclimation began 20 days after sowing. Plants were transferred to a growth chamber set to 4 °C/2 °C (day/night) with a 9 h/15 h (light/dark) photoperiod and an irradiance of 250 µmol m^−2^ s^−1^, and maintained under these conditions for 21 days. Following the cold acclimation period, plants were subjected to active de-acclimation for 7 days in conditions mimicking a mid-winter warm spell: 12 °C/5 °C (day/night) with a 10 h/14 h (light/dark) photoperiod.

The physiological measurements were recorded at two time points: (1) CA (after 21 days of cold acclimation), and (2) DA (after 7 days of de-acclimation following the cold-acclimation period).

### 4.3. Genotyping

Genomic DNA was extracted from young leaf tissues of the 188 barley accessions using the DNeasy Plant Mini Kit (Qiagen, Hilden, Germany), following the manufacturer’s instructions. DNA quality and concentration were assessed using a UV-Vis Q5000 spectrophotometer (Quawell, San Jose, CA, USA). High-quality DNA samples were genotyped using Diversity Arrays Technology sequencing (DArTseq; https://www.diversityarrays.com/products-and-services/applications/ (accessed on 30 September 2022) and Single Nucleotide Polymorphism (SNP) profiling, following procedures previously described by [56]. Quality parameters, including call rate, polymorphic information content (PIC), and marker reproducibility, were selected according to the same protocol.

### 4.4. Association Analysis

Mixed Linear Model (MLM) association mapping was performed using TASSEL v5.2.188 software [57]. The MLM included a kinship matrix (K) computed via the centered Identity-By-State (IBS) method and population structure (Q) estimated through principal component analysis (PCA). Genotypic data were imputed using LD KNNi and filtered with a minor allele frequency (MAF) threshold of 0.05. Default TASSEL settings were applied for all other parameters. Marker–trait associations were assessed genome-wide, and significant markers were selected based on Manhattan plots using a significance threshold of *p* ≤ 0.01. Probability values from the MLM were adjusted using false discovery rate (FDR) according to the procedure by Benjamini and Hochberg (1995) [58] with α = 0.05.

## Figures and Tables

**Table 1:** List of selected markers significantly associated with key traits in winter barley in both de-acclimated (DA) and cold-acclimated (CA) states, identified by genome-wide association studies (GWASs) using the mixed linear model (MLM).

MARKER	TYPE	CHR	POSITION	TRAITS ASSOCIATED WITH DA								TRAITS ASSOCIATED WITH CA							
3264267	DA DART	1H	72428160	ABS/CS	DI_0_/CS	ET_0_/CS	Survival rate	TR_0_/CS				ABS/CS	DI_0_/CS	DI_0_/RC					
3270144	DA DART	7H	639580297	ABS/CS	ABS/RC	Survival rate	TR_0_/RC					TR_0_/RC							
3271448	DA DART	7H	639653526	ABS/CS	ABS/RC	Survival rate	TR_0_/RC					TR_0_/RC							
3272400	DA DART	2H	132061889	DI_0_/CS	F_v_/F_m_	RC/CS_m_	Survival rate					ABS/CS	ABS/RC	DI_0_/CS	DI_0_/RC	ET_0_/CS	TR_0_/CS	TR_0_/RC	
3397788	DA DART	1H	371321664	ABS/CS	ABS/RC	DI_0_/CS	ET_0_/CS	Survival rate	TR_0_/CS	TR_0_/RC		PI_(total)_							
3662522	DA DART	1H	447746864	ABS/CS	ABS/RC	PI_(ABS)_	Survival rate					DI_0_/CS	DI_0_/RC						
9773215	DA DART	1H	447502769	ABS/CS	ABS/RC	DI_0_/CS	DI_0_/RC	PI_(ABS)_	Survival rate			ABS/CS	ABS/RC	DI_0_/CS	DI_0_/RC				
11400277	DA DART	7H	639599562	ABS/CS	ABS/RC	DI_0_/CS	DI_0_/RC	PI_(ABS)_	Survival rate	TR_0_/CS	TR_0_/RC	ABS/CS	ABS/RC	DI_0_/CS	DI_0_/RC	ET_0_/RC	Survival rate	TR_0_/CS	TR_0_/RC
100009417	DA DART	7H	642200021	ABS/CS	ABS/RC	DI_0_/CS	Survival rate	TR_0_/CS				TR_0_/CS	TR_0_/RC						
6437688	DA SNP	4H	332990147	ABS/RC	DI/CS	DI_0_/RC	ET_0_/RC	Survival rate				PI_(total)_							

## Data Availability

The datasets generated and/or analyzed during the current study are available from a public database. Raw DArTseq and SNP data are available from University of Agriculture in Krakow repository (DOI:10.15576/REPOURK/2026.1.2); link to download the research data: https://repo.ur.krakow.pl/info/researchdata/URK24b5d4af4d5f498db36cc9982d5515fb/ (accessed on 19 February 2026).

## Supplementary Materials

The following supporting information can be downloaded at https://www.mdpi.com/article/10.3390/ijms27062759/s1.

## Abbreviations

The following abbreviations are used in this manuscript:

GWAS	Genome-wide association study
MLM	Mixed-linear model
SNP	Single nucleotide polymorphism
DArTseq	Diversity Arrays Technology sequencing
CA	Cold-acclimated
DA	De-acclimated
CHR	Chromosome
