# Supplementary material for: Genome-Wide Association Study (GWAS) for Freezing and De-Acclimation Tolerance in Polish Winter Barley
Source: Int J Mol Sci. 2026 Mar 18;27(6):2759. doi: 10.3390/ijms27062759 (PMC13026713; doi:10.3390/ijms27062759)
Supplement: Supplementary file 1 [file ijms-27-02759-s001.zip › Supplement_Table_S2_Chlorophyll fluorescence parameters details.pdf]

Table S2. Formulae and glossary of terms used by the OJIP-test.

(In the present study**,** parameters are modified after Strasser et al. 2004).

<u>Data extracted from the recorded fluorescence transient OJIP</u>	
F_t	fluorescence at time t after onset of actinic illumination
$F_{50\mu s}$ or $F_{20\mu s}$	minimal reliable recorded fluorescence, at 50 µs with the PEA-<br>or 20 µs with the Handy-PEA-fluorimeter
$F_{300\mu s}$	fluorescence intensity at 300µs
$F_J \equiv F_{2ms}$	fluorescence intensity at the J-step (2 ms) of OJIP
$F_I \equiv F_{30ms}$	fluorescence intensity at the I-step (30 ms) of OJIP
F_P	maximal recorded fluorescence intensity, at the peak P of OJIP
<u>Fluorescence parameters derived from the extracted data</u>	
$F_0 \cong F_{50\mu s}$ or $\cong F_{20\mu s}$	minimal fluorescence (all PSII RCs are assumed to be open)
F_M (= F_P)	maximal fluorescence, when all PSII RCs are closed (equal to F_P when the actinic light intensity is above 500 µmol photons m^{-2} s^{-1}<br>and provided that all RCs are active as Q_A reducing)
$F_V \equiv F_M - F_0$	maximal variable fluorescence
$V_t \equiv F_v/F_V \equiv (F_t - F_0)/(F_M - F_0)$	relative variable fluorescence at time t
$M_0 \equiv [(\Delta F/\Delta t)_0]/(F_M - F_{50\mu s})$<br>$\equiv 4(F_{300\mu s} - F_{50\mu s})/(F_M - F_{50\mu s})$	approximated initial slope (in ms^{-1}) of the fluorescence transient normalised on the maximal variable fluorescence F_V
<u>Specific energy fluxes</u> (per Q_A-reducing PSII reaction center - RC)	
$ABS/RC = M_0 (1/V_J)(1/\phi_{Po})$	absorption flux (of antenna Chls) per RC
$TR_0/RC = M_0 (1/V_J)$	trapped energy flux (leading to Q_A reduction) per RC
$ET_0/RC = M_0 (1/V_J)\psi_{Eo}$	electron transport flux (further than Q_A) per RC
$DI_0/RC = (ABS/RC) - (TR_0/RC)$	dissipated energy flux, per RC
<u>Quantum yields and efficiencies</u>	
$\phi_{Po} \equiv TR_0/ABS = Fv/F_M$	maximum quantum yield for primary photochemistry
$\psi_o \equiv ET_0/TR_0 = (1-V_J)$	efficiency/probability for electron transport (ET), i.e. efficiency/probability that an electron moves further than Q_A^-
$\phi_{Eo} \equiv ET_0/ABS = [1-(F_0/F_M)]\psi_{Eo}$	quantum yield for electron transport (ET)
$\phi_{Ro} \equiv RE_0/ABS = [1-(F_0/F_M)]\psi_{Eo}\,\delta_{Ro}$	quantum yield for reduction of end electron acceptors at the PSI acceptor side (RE)

Phenomenological fluxes	
ABS/CS = *F*o or ABS/CSM = *F*M<br>TR_0/CS = ΦPo·(ABS/CS)<br>ET_0/CS = ΦPo·Ψo·(ABS/CS)<br>DI_0/CS = (ABS/CS) – (TRo/CS)	absorption per excited cross-section<br>trapping per excited cross-section<br>electron transport per excited cross-section<br>dissipated energy flux per excited cross-section
<u>Performance indexes</u> (products of terms expressing partial potentials at steps of energy bifurcations)	
$PI_{ABS} \equiv [\gamma_{RC}/(1-\gamma_{RC})].[\phi_{Po}/(1- \phi_{Po})].[\psi_o/(1- \psi_o]$	performance index (potential) for energy conservation from<br><br>exciton to the reduction of intersystem electron acceptors
$PI_{total} \equiv (PI_{ABS}).(\delta_{Ro}/1- \delta_{Ro})$	performance index (potential) for energy conservation from<br><br>exciton to the reduction of PSI end acceptors
Subscript "0" indicates that the parameter refers to the onset of illumination	
